# Acute focal bacterial nephritis in a renal allograft

**DOI:** 10.1590/2175-8239-JBN-2020-0019

**Published:** 2020-12-11

**Authors:** Luísa Pereira, Joana Marques

**Affiliations:** 1 Centro Hospitalar Universitário do Algarve, Departamento de Nefrologia, Faro, Portugal.; 2 Centro Hospitalar Universitário Lisboa Central, Departamento de Nefrologia, Lisbon, Portugal.

## Case report and descriptive figure legend

Acute focal bacterial nephritis (AFBN) is a rarely diagnosed localized non-liquefactive bacterial infection of the kidney^1^. Due to the non-specific clinical presentation of this entity, correct diagnosis can be challenging.

We present the case of a 38-year-old female with end stage renal disease secondary to atypical hemolytic uremic syndrome (aHUS) who was submitted to kidney transplantation with prophylactic eculizumab administration. Allograft function was within the normal range since the 2^nd^/3^rd^ day posttransplant. On day 12, a protocol kidney biopsy was performed, showing an endoluminal thrombus, leading to an increase of eculizumab dose. Three months after kidney transplantation she presented with a 2-day history of fever, nausea, vomiting, and diarrhea. She denied abdominal pain, dysuria, urinary output reduction, or other symptoms. Clinical examination revealed slight allograft tenderness and fever up to 40ºC.

Laboratory investigation revealed leucopenia, elevated c-reactive protein, and *de novo* allograft dysfunction. An empiric intravenous antibiotic therapy was initiated and further radiological imaging was scheduled.

**Figure 1:**
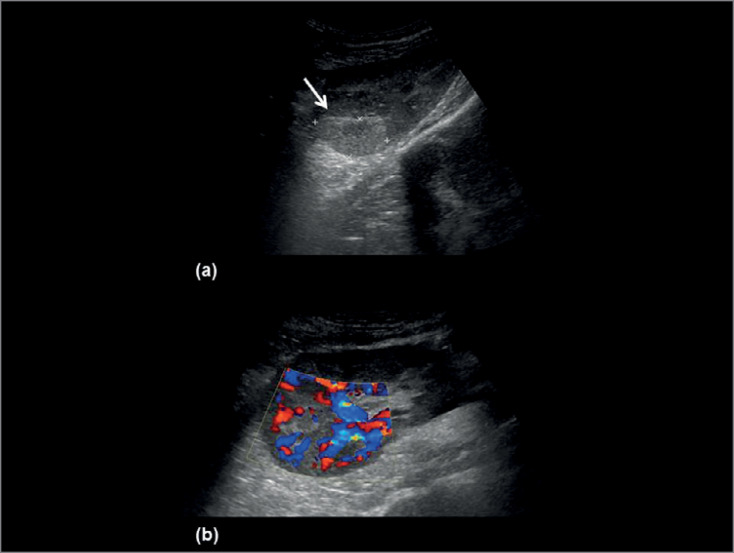
Panel A. Doppler ultrasound (DUS) showing rounded-shaped focal hyperechoic lesions (arrow); Panel B. Doppler ultrasound showing no perfusion abnormalities.

Doppler ultrasound (DUS) showed rounded-shaped focal hyperechoic lesions, the largest with 40 mm on the major axis (Figure 1 - panel A, arrow) without perfusion abnormalities (Figure 1 - panel B), indicating the possibility of iatrogenic bleeding after biopsy.

**Figure 2:**
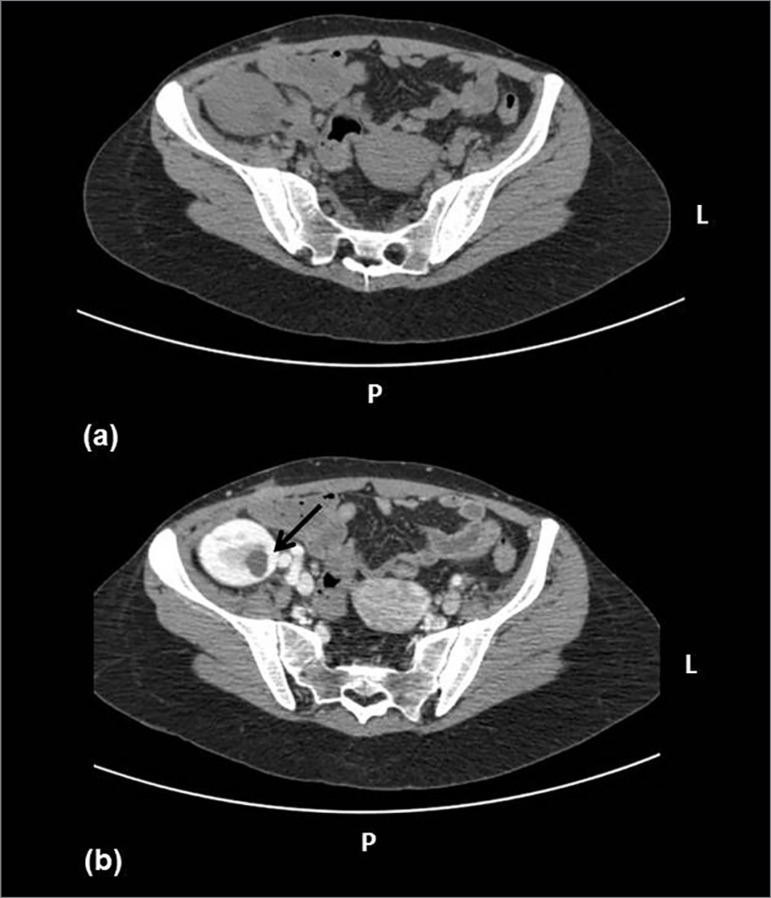
Panel A. Non-enhanced CT scan showing no attenuation area in the allograft. Panel B. Enhanced CT scan showing disclosed oval and wedge-shaped decreased enhancement areas, without cortical rim sign (outer cortical contrast uptake) in the upper third of the allograft (arrow).

In order to clarify the diagnosis she was submitted to a contrast enhanced computerized tomography (CT) scan, which disclosed oval and wedge-shaped decreased enhancement areas, without cortical rim sign (outer cortical contrast uptake) in the upper third of the allograft (Figure 2 - panel B, arrow), consistent with AFBN. Blood and urine cultures isolated a multi-sensitive *Escherichia coli*, and antibiotic therapy was switched according to antibiotic susceptibility testing results.

The patient's post treatment course was uneventful, and two weeks later, follow-up DUS returned to normal showing resolution of parenchymal lesions. She was discharged on the 16^th^ day after admission in good clinical condition.

AFBN, previously known as acute lobar nephronia, has been recognized as a localized infection of the kidney, representing the progression of acute pyelonephritis although without abscess formation, most commonly caused by *Escherichia coli* ^2^. Ultrasound shows circular either hypo- or hyperechogenic, hypoperfused parenchyma lesions, which may be misdiagnosed as a renal abscess, a tumor, or a renal infarction usually requiring CT to elucidate the focal pattern of kidney lesion^1^^,^ 2. Symptoms can resemble acute pyelonephritis but can also be non-specific including nausea, headache, and abdominal guarding, mimicking other clinical entities such as acute abdomen. As in this clinical case, we need to keep in mind that patients with aHUS may have an increased risk of infection even prior to treatment and that Eculizumab blocks activation of C5b (and thus MAC formation), which is expected to lead to higher susceptibility to bacterial infection^4^^,^ 5.

Renal ultrasonography or CT scanning has been found to demonstrate the focal nature of AFBN more distinctly with nephrogram diminution of the lesion^6^. Differentiation of AFBN from an abscess is dependent on clear characterization of the lesion's density, shape, and margins, although there can be overlap among these. Difficult cases may eventually require diagnostic/therapeutic needle aspiration to conclude whether or not the lesion is fluid-filed^6^^,^ 7. Accurate interpretation of both clinical and radiological findings is crucial to differentiate AFBN from uncomplicated pyelonephritis, renal abscess, and pyonephrosis, which may require different treatment strategies with more extensive interventions^8^. As soon as localized uni- or multifocal lesions are detected, the term acute focal (or multifocal) bacterial nephritis should be used implicating a higher risk for complicated disease course and redundant interventions. The objective is not to distinguish all cases of AFBN from acute pyelonephritis but to identify serious and atypical courses of AFBN requiring special attention and prolonged treatment^2^ Web of Science. The absence of cortical rim sign can be helpful in differentiating AFBN from renal infarction^7^. Invasive diagnostic and therapeutic procedures should be limited as the majority of cases respond well to conservative treatment, although in rare cases nephrectomy might be necessary due to non-response to medical treatment^3^.
